# Red-light phenotype in a marine diatom involves a specialized oligomeric red-shifted antenna and altered cell morphology

**DOI:** 10.1038/s41598-017-12247-0

**Published:** 2017-09-20

**Authors:** Miroslava Herbstová, David Bína, Radek Kaňa, František Vácha, Radek Litvín

**Affiliations:** 1 Institute of Plant Molecular Biology, Biology Centre, Czech Academy of Sciences, Branišovská 31, 37005 České Budějovice, Czech Republic; 2 Faculty of Science, University of South Bohemia, Branišovská 1760, 37005 České Budějovice, Czech Republic; 3 Institute of Microbiology, Algatech Centre CAS, Opatovický mlýn, 379 81 Třeboň, Czech Republic

## Abstract

Diatoms greatly contribute to carbon fixation and thus strongly influence the global biogeochemical balance. Capable of chromatic acclimation (CA) to unfavourable light conditions, diatoms often dominate benthic ecosystems in addition to their planktonic lifestyle. Although CA has been studied at the molecular level, our understanding of this phenomenon remains incomplete. Here we provide new data to better explain the acclimation-associated changes under red-enhanced ambient light (RL) in diatom *Phaeodactylum tricornutum*, known to express a red-shifted antenna complex (F710). The complex was found to be an oligomer of a single polypeptide, Lhcf15. The steady-state spectroscopic properties of the oligomer were also studied. The oligomeric assembly of the Lhcf15 subunits is required for the complex to exhibit a red-shifted absorption. The presence of the red antenna in RL culture coincides with the development of a rounded phenotype of the diatom cell. A model summarizing the modulation of the photosynthetic apparatus during the acclimation response to light of different spectral quality is proposed. Our study suggests that toggling between alternative organizations of photosynthetic apparatus and distinct cell morphologies underlies the remarkable acclimation capacity of diatoms.

## Introduction

Widespread and diverse diatoms represent an ecologically important group of primary producers among heterokont algae^1–4^. Marine diatoms are found both (i) free-living in the open ocean as a prominent component of phytoplankton, and (ii) biofilm-forming in benthic habitats such as coastal waters, estuaries and rock pools^5,6^. Their global abundance depends on a strong ability to adapt to the changing environment including variation of light intensity and spectral quality along the water column^7–12^. Response of photosynthetic organisms to changes in the spectral composition of the ambient radiation was discussed under the terms of chromatic adaptation theory as early as in the 1800’s by Engelman^13^. Both the term (chromatic) ‘adaptation’ and ‘acclimation’ can be found in literature referring to these processes. We are of the opinion that ‘acclimation’ is preferable to refer to a short-term physiological response of an organism to environmental conditions while ‘adaptation’ should be reserved to description of an evolutionary process. Thus, the term chromatic acclimation will be used in the following text.

The key role in chromatic acclimation (CA) is played by the light-harvesting component of the photosynthetic machinery, manifesting in changes of the pools of photosynthetic pigments^14^. With the advent of genomic data^15,16^ and progress of proteomic methods it became possible to achieve functional classification of the proteins of the light-harvesting apparatus as well as to study the response of the transcriptome and proteome to environmental variables, including different light regimes^9,17–22^.

The light-harvesting apparatus of the model diatom *Phaeodactylum (P*.*) tricornutum*, comprises several groups of light-harvesting proteins^23^: i) a set of *Lhcr* proteins that form the antenna of the photosystem I (PSI), coded for by 14 genes; ii) 4 stress-related *Lhcx* proteins; and iii) the largest group of 17 *Lhcf* proteins that includes the principal light-harvesting complex (LHC) of diatoms, the fucoxanthin chlorophyll *a*/*c*–binding proteins (FCPs). The reason for the high number of LHCs and their physiological roles are still a matter of ongoing research.

One intriguing aspect of CA is a formation of red-shifted forms of chlorophyll (Chl) *a* induced in the algal cultures grown under red-enhanced illumination^24,25^. The absorption of these Chls extends above 700 nm and their presence is conspicuously indicated by a strong far-red fluorescence at room temperature, originally labelled as F710^26^. Fujita & Ohki^26^ localized the F710 emitter generally in the PSII-related pool of LHC’s. However, fragility of the F710 emitting antenna complex prevented its detailed spectroscopic and biochemical characterization. Moreover, bulk spectroscopy on algal cultures expressing the red-shifted complex did not provide information whether the cultures grown under red light and expressing the red-antenna were homogeneous, and truly formed a well-defined phenotype. Recently, the presence of the red Chl *a* forms in *P*. *tricornutum* was linked to the expression of a specialized light-harvesting protein, Lhcf15^25^. Furthermore, the red-light-associated phenotype of *P*. *tricornutum* was later found to extend to altered supramolecular organization of photosystem I (PSI) and thylakoid ultrastructure^27^.

In this study, we have succeeded in obtaining the purified red-light induced Chl *a* F710 antenna complex in a form that retained the red-shifted Chl *a*. Thus, for the first time, a precise analysis of its protein composition, steady state spectroscopic properties at room temperature and the oligomeric state was achieved.

As the major component of phytoplankton, diatoms play a significant role in global geochemical cycles. Understanding the adaptation strategies that allow diatoms to thrive in highly variable aquatic environments is therefore of paramount importance. Here we show that exposure to light conditions characterised by depletion of photosynthetically active radiation in the blue-green range with accompanying relative increase of the red-far red component leads to development of a specific phenotype in the model diatom *P*. *tricornutum*. This phenotype is characterized both by a distinct cell morphology, and by production of a specific antenna polypeptide that assembles into oligomeric complexes harbouring the far-red absorbing species of Chl *a*. This complex set of traits provides a striking example of chromatic acclimation in algae of the red lineage.

## Results

### Purification of the antenna complexes in a native state

**Figure 1:**
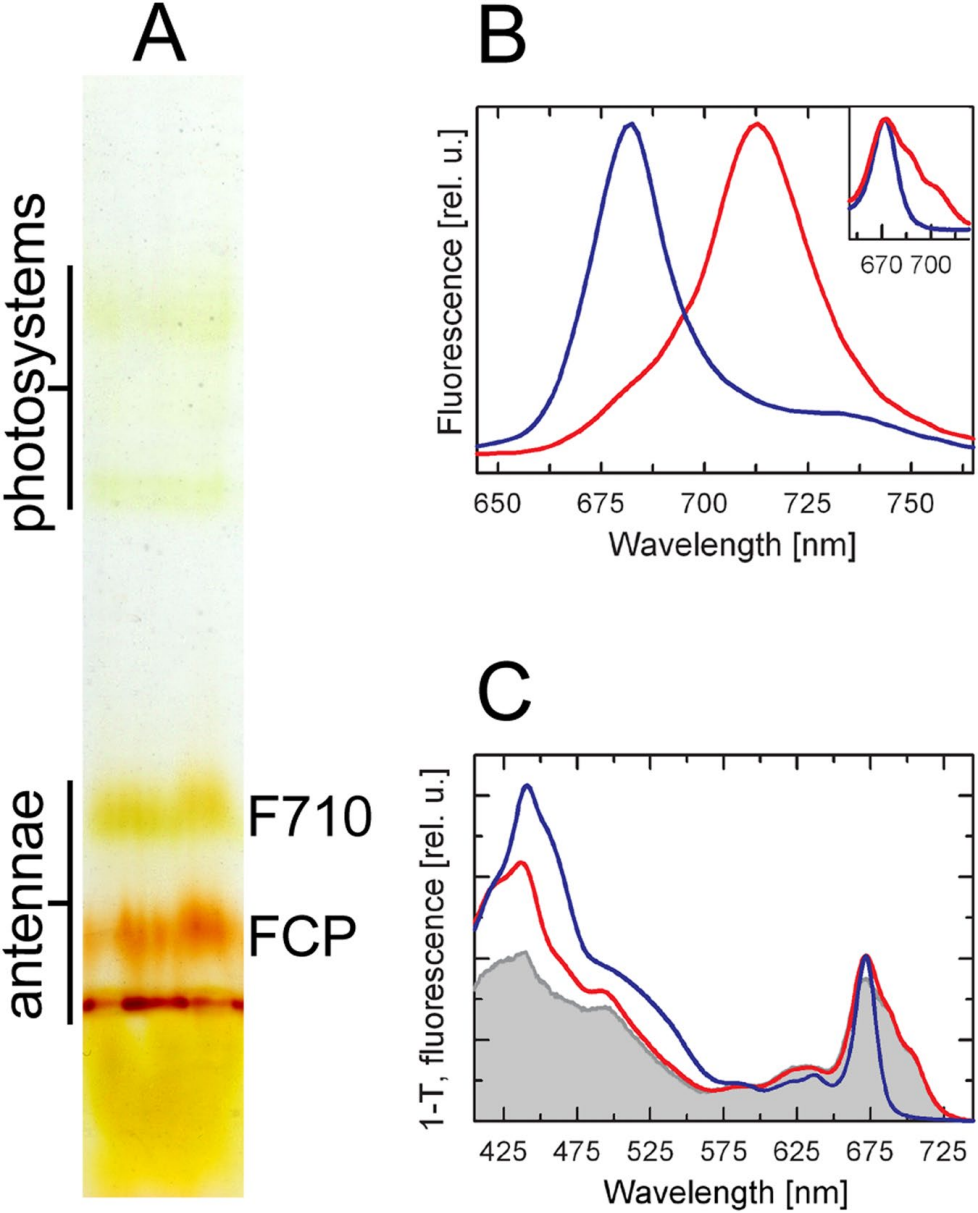
Biochemical and biophysical characterization of the Lhcf protein-based oligomeric antenna complexes in the RL-adapted *P*. *tricornutum*. **(A)** CN-PAGE of the gel filtration fraction 3 (cf. chromatographic profile in Supplementary Fig. S1B) resulted in separation of two oligomeric and one trimeric antenna band on the gel, (**B**) Room temperature fluorescence emission spectra of the F710 (*red line*) and FCP oligomeric antenna band (*blue line*), and (*Inset*) Q_y_ regions of the absorption spectra of the F710 (*red*) and the oligomeric FCP gel band (*blue*). **(C**) Fluorescence excitation spectrum of the F710 band (*grey*, detected at 730 nm) and 1-T absorption spectra of the F710 (*red*) and oligomeric FCP band (*blue*). Fluorescence spectra in (**B**) were normalized at their maxima, 1-T spectra were normalized at their Q_y_ maxima.

Colourless native electrophoresis (CN-PAGE) of the gel filtration fraction 3 (Suppl. Fig. S1B) from the red-light (RL) culture yielded three well-resolved antenna complexes of different apparent molecular masses as well as two weak green-coloured bands of photosystem contaminants (Fig. 1A). Referring to earlier works^28,29^, the lowermost antenna band can be interpreted as a trimer and the upper two as oligomers of different size. Judging from the brown colour of the lower two antenna bands (the trimer and the smaller oligomer), these were ascribed to the forms of FCP antenna. Consequently, the uppermost, greenish antenna band was interpreted as the F710 antenna. This quick assessment based on the comparison to antenna complexes of *C*. *velia* ^24^ was confirmed by the spectroscopic measurements, as detailed in the following paragraphs.

### Spectral heterogeneity in the antenna systems

The steady-state absorption and fluorescence spectroscopy clearly distinguished the F710 antenna from the FCP, as shown in Fig. 1B,C. At room temperature, the FCP oligomer emitted a single peak with a maximum at ~682 nm. As shown in Suppl. Fig. 2, the emission spectrum of the FCP trimer was similar to that of the oligomer except for a ~2-nm blue shift of the emission maximum. On the contrary, the emission spectrum of the F710 antenna exhibited a strongly red-shifted maximum peaking at ~713 nm (Fig. 1B) accompanied by a shoulder on the blue edge of the main emission band.

Consistent with the different emission properties, the absorption spectra of the antenna bands showed a major difference in the Chl *a* Q_y_ region, as seen in Fig. 1B inset. The chlorophyll Q_y_ peak (maximum at ~672 nm) of the F710 antenna complex was markedly extended towards the far-red region, with two pronounced shoulders at ~690 nm and ~705 nm, whereas the FCP antenna showed a narrow band peaking at ~672 nm. As expected from the colour difference between the CN-PAGE bands, the absorption spectra of the F710 antenna showed lower contribution of the red-shifted carotenoids (fucoxanthin) and Chl *c*, compared to the FCP band (Fig. 1C). Instead, the blue-green region of the spectrum was dominated by a carotenoid absorption band at ~490 nm (likely a 0–0 vibronic transition of diadinoxanthin). In agreement, pigment analysis of F710 antenna extracted from CN-PAGE by HPLC (see Methods) indicated, per 100 Chl *a*, 10 Chl *c*_2_, 58 fucoxanthins and 8 diadinoxanthins, compared to about 31 Chl *c*_2_, 115 fucoxanthins and <1 diadinoxanthin in FCP. Corresponding stoichiometries were also obtained by fits of absorption spectra of bands from CN-PAGE gels (Suppl. Fig. 3).

Fluorescence excitation spectrum of the F710 band (Fig. 1C) recorded at 730 nm showed overall high efficiency (>80%) of the excitation transfer from carotenoids to the red forms of Chl *a*. Since the CN-PAGE gel contains local inhomogeneities that contribute to light scattering, thus increasing the optical density of the sample in the shortwave (blue) region, the measured efficiency of energy transfer from carotenoids to Chl can be considered to be the lower limit.

All measurements described above were performed on gel strips excised from CN-PAGE gels. Interestingly, the stability of the F710 oligomer was enhanced by embedding in the CN-PAGE gel. The antenna retained the red-shifted spectral features, even after freezing and thawing of the gel, in striking contrast to the purified sample in solution, or even to whole cells or to fresh thylakoid preparation^25,26^.

### The F710 antenna oligomer consists exclusively of the Lhcf15 protein

**Figure 2:**
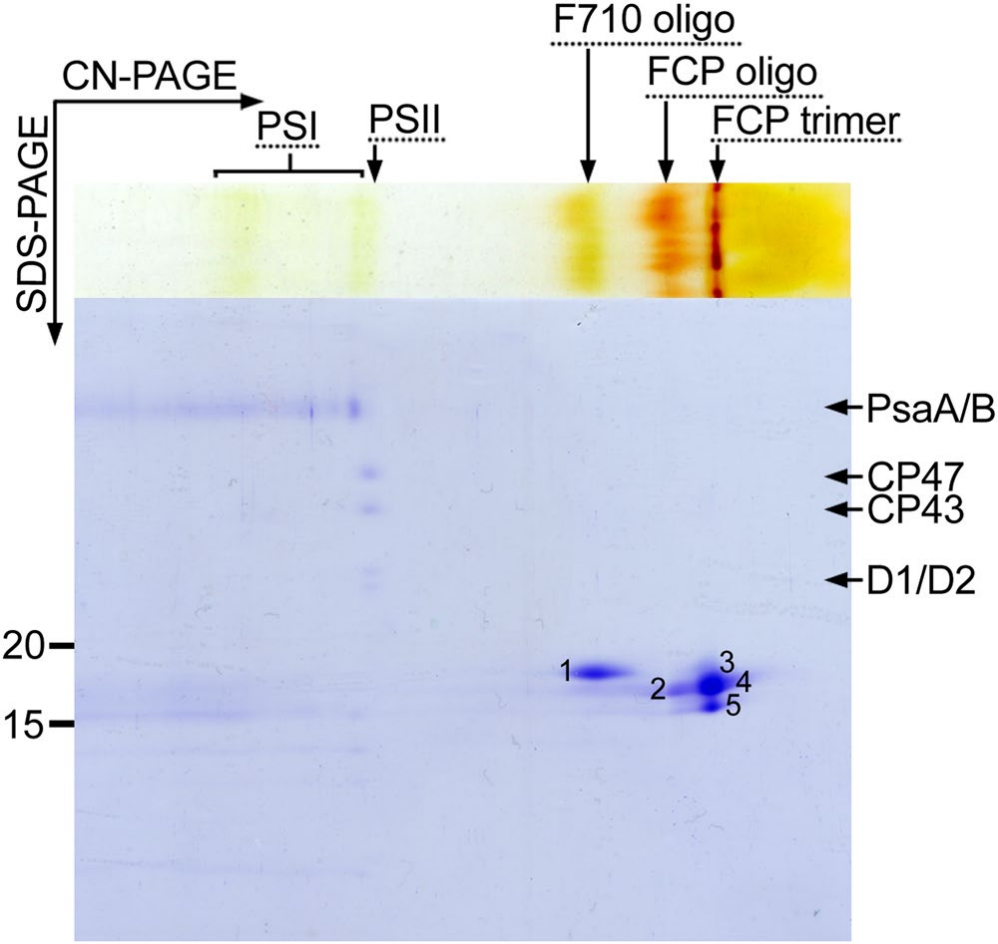
Two-dimensional protein composition analysis of the antenna complexes in the RL-adapted *P*. *tricornutum*. Spots 1–5 corresponding to antenna proteins were analyzed by mass spectrometry (see Table 1).

We have recently proposed the Lhcf15 protein as the most likely candidate required for the longwave emission^25^. Here, the combination of 2D gel separation (Fig. 2) with mass spectrometry analysis (Table 1, Suppl. Table 1) allowed us to determine the exact protein composition of the F710 antenna oligomer. Only the Lhcf15 protein was repeatedly found in spot 1 corresponding to the F710 band on the CN gel (Fig. 2). Thus Lhcf15 presents the sole component of the F710 antenna.

**Table 1:** Protein composition of the oligomeric F710 and FCP, and trimeric FCP antenna complexes in the RL-adapted *P*. *tricornutum* identified by tandem MS analysis. Identification numbers of spots from Fig. 2 and number of matches out of five biological replicates are indicated. See Suppl. Table 1 for a list of identified peptides.

F710 oligomer			FCP oligomer			FCP trimer		
spot #	Protein	# matches	spot #	Protein	# matches	spot #	Protein	# matches
1	Lhcf15	5	2	Lhcf1	5	5	Lhcf1	5
			2	Lhcf2	5	5	Lhcf2	5
			2	Lhcf3/4	5	4	Lhcf3/4	5
			2	Lhcf5	5	5	Lhcf5	5
			2	Lhcf6/7	4	4	Lhcf6/7	3
			2	Lhcf8	3	4	Lhcf8	5
			2	Lhcf9	5	3	Lhcf9	3
			2	Lhcf10	5	4	Lhcf10	5
			2	Lhcf11	5	4	Lhcf11	5
						3	Lhcf15	2

We also analyzed the protein composition of the FCP antenna oligomer. As listed in Table 1, only Lhcf proteins other than Lhcf15 were found in the spots corresponding to the oligomeric FCP band on the CN gel (Fig. 2). All of the Lhcf proteins forming the FCP oligomeric antenna were also detected in the spots representing the trimeric FCP assembly, which in addition included Lhcf15. This likely indicates that Lhcf15, like many other antenna proteins of the LHC superfamily, forms also smaller assemblies, presumably trimers. However, the absence of the far-red emission signal in the fluorescence spectrum of the trimeric antenna band (Suppl. Fig. S2) showed that the trimeric assembly of the Lhcf15 is insufficient for its long-wave function. The trimers may represent an intermediate in the assembly process leading to the oligomeric complexes.

### RL cells possess a distinct morphology

**Figure 3:**
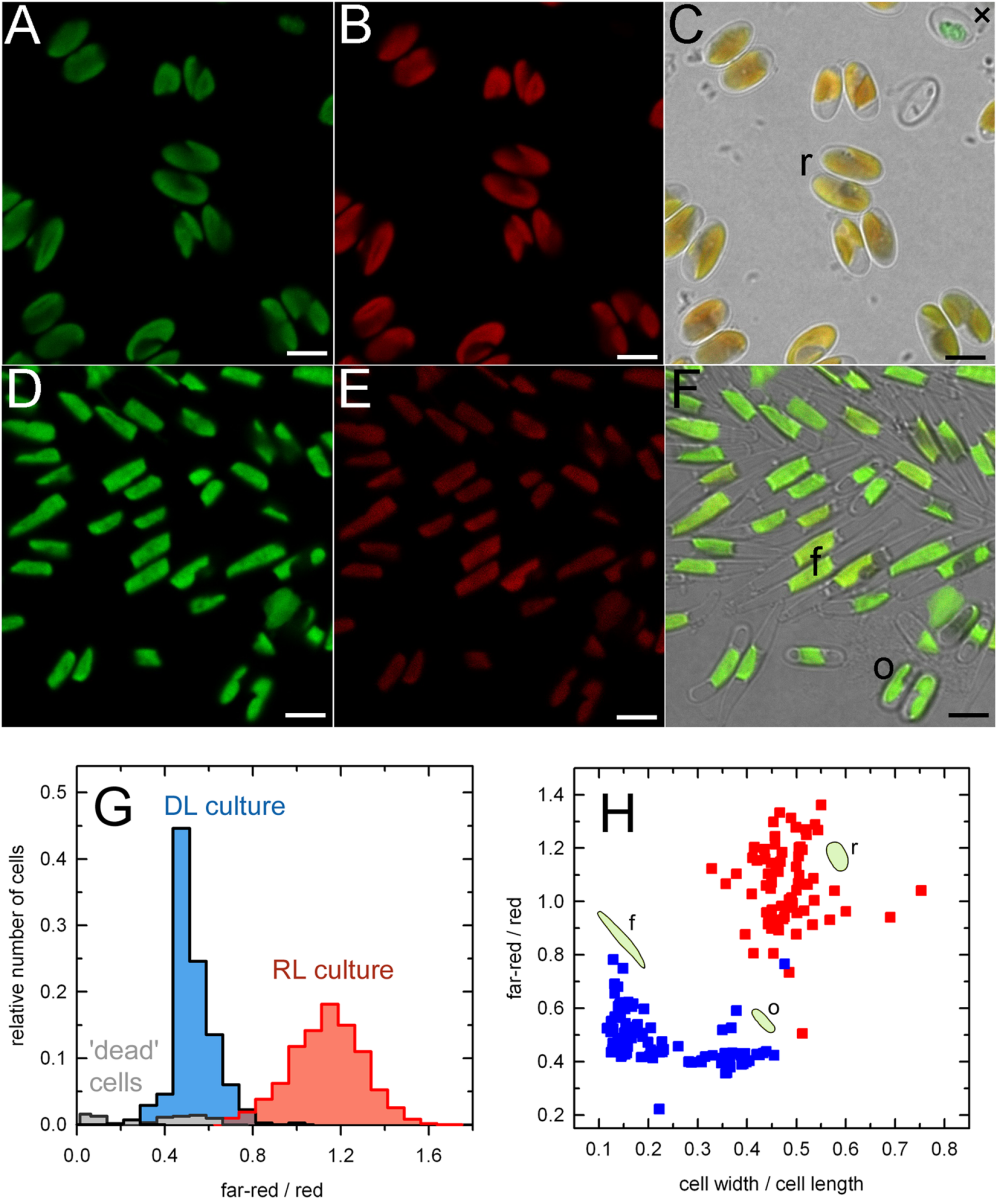
Confocal microscopy image analysis of the *P*. *tricornutum* cells grown under the RL (**A**,**B**,**C**) and DL (**D**,**E**,**F**) conditions. Images showing emission signals of chlorophyll autofluorescence recorded in a dual channel setup in the wavelength ranges of red (650–696 nm) (**A**,**D**) and far-red (705–735 nm) (**B**,**E**) bands. Composite images on the right (**C**,**F**) were produced by merging of the red and far-red channels and images concurrently detected in transmitted light mode. Scale bars correspond to 5 µm. Typical examples of the cell morphotypes found in our cultures are labelled as follows: f, fusiform; o, oval; r, rounded. (**G**) A histogram of the far-red / red emission ratios of the DL (*blue*) and RL (*red*) culture. More than 300 individual cells from each culture condition were analyzed (see Material and Methods). The grey plot indicates aberrant, presumably dead, cells such as the one labelled with a cross (**×**) in panel C. Quantitative characterization of the morphological variability of the cells acclimated to the RL and DL conditions and its relationship to the spectroscopic characteristics is shown in (**H**). Morphotypes are indicated with cartoons and letters as in panels C and F.

In order to characterize the distribution of the F710 antenna type in the RL cell population in detail, we used confocal laser scanning microscopy with a narrow plane of focus and multi-channel recording. In this experiment, we used a day-light (DL) culture as a comparative sample to the RL culture as the light-harvesting apparatus of the DL-grown *P*. *tricornutum* utilizes FCP type of antenna only. The emission signals of chlorophyll autofluorescence excited with a 488-nm laser were detected concurrently in the red region (below 696 nm) and the far-red region (above 705 nm). In Fig. 3, the red emission channel is shown in green and the far-red channel in red colour. The images showed the distribution of the red and far-red emission signals to be even within the method resolution throughout the plastids in both DL and RL cultures (Fig. 3A,B and D,E). The difference in the contribution of the red and far-red fluorescence to the overall emission of the cells from the RL and DL cultures is shown in the composite images (Fig. 3, panels C and F). Statistical characterization of the spectroscopic parameters in both cultures (Fig. 3G) was performed in more than 300 individual cells from each culture condition. The histograms in Fig. 3G show the relative abundance of the cells exhibiting given ratio of far-red / red fluorescence emission in the DL (blue) and RL (red) culture. The increased far-red emission was universally present in cells from the RL culture and both cultures were well defined and quite homogeneous in their properties, as seen from the well-separated, unimodal distributions of the fluorescence ratio.

In addition to the spectroscopic characteristics, confocal imaging showed variability in morphology of the RL and DL cells (Fig. 3C and F). Following De Martino *et al*.^30^, we denote three cell morphotypes observed in our work as (i) fusiform (spindle-shaped), (ii) oval, and (iii) rounded. The triradiate cell shape was not observed here in an appreciable amount. Quantitatively, the morphotypes could be characterized by the width-to-length ratio of the bright-field images of the cells as shown in Fig. 3H. Only the rounded morphotype was observed in the RL cells regardless of the age of the culture (Fig. 3C). In contrast, the DL culture was a mixture of fusiform and oval morphotypes (Fig. 3F and H) with the latter increasing in abundance with the age of the culture. As shown in the plot in Fig. 3H, we observed that there was only a minor difference in the far-red/red ratio between the fusiform and oval cells in the DL culture. On the other hand, the far-red emission was clearly a defining feature of the rounded cells found in the RL culture (Fig. 3G).

Consistently with previous reports on chromatic acclimation^26,31^, changes in both the spectroscopic properties (F710) and the morphology of the cells were reversible on the time scale of days following transition from RL to DL conditions, and vice versa.

To evaluate the effect of RL more rigorously, the growth rate of the DL culture was lowered to match the RL culture (growth rate 0.19 ± 0.05 day^−1^). This was achieved by decreasing the incident irradiance to ~7 µmol photons m^−2^ s^−1^ while maintaining the DL spectral composition. The resulting growth rate of this DL culture was 0.16 ± 0.05 day^−1^ (mean ± SD of three biological replicates). The emission spectra of the culture were recorded and the cell morphology was also analyzed. As shown in Suppl. Fig. S4A, decreased growth rate of the DL culture was accompanied by about 5 nm redshift of the fluorescence emission maximum and broadening on the red edge of the emission spectrum. However, up to 25th day of experiment, the emission spectrum did not develop a distinct F710 peak. Moreover, although the slowly growing culture showed increased proportion of oval cells, it did not exhibit a full transition towards rounded morphotype observed in typical RL culture after equal time (Suppl. Fig. S4B). Thus, even under condition of identical growth rates, the main differences between the DL and RL were maintained, supporting the effect of incident light quality not only on the development of the red-shifted antenna but also on the morphology of *P*. *tricornutum*.

## Discussion

Recent studies have shown that when grown under red-enhanced illumination, *P*. *tricornutum* develops a phenotype characterized by the presence of low-energy Chl *a* forms responsible for the pronounced red-shift of the emission maximum of the algal culture^25,26^. This spectroscopic signature is tied to the presence of the antenna protein Lhcf15. Beyond the changes in the protein composition of the light-harvesting system, features of the RL-grown cells included altered photosystem distribution and expansion of the thylakoid membrane system^27^.

The data presented here complete the picture of the red-light acclimation syndrome of *P*. *tricornutum*. Firstly, our study establishes the Lhcf15 protein as the sole building unit of the red-shifted antenna complex. This protein forms an oligomeric assembly that is required to maintain the spectral properties of the F710 antenna. Although studies on the light-harvesting apparatus of *P*. *tricornutum* originally reported that the major FCP antenna consisting of Lhcf proteins was organized into trimeric complexes^28,32,33^, other works have shown that the Lhcf antenna can occur at different degrees of oligomerization^34,35^. We obtained similar results for the Lhcf15 protein that was found here in both trimeric and oligomeric states. However, only the oligomeric form exhibited the characteristic far-red emission, in agreement with earlier studies of the red-shifted antenna in the alveolate *Chromera velia* ^24,31^. Based on these findings, we consider the oligomer to be the native form of the red antennae present in the thylakoid membrane of *P*. *tricornutum* whereas the trimers represent an intermediate step of the oligomer assembly or result from its disintegration.

Despite their sequence homology, the Lhcf proteins forming the major FCP antenna and the red-light induced F710 antenna bind spectrally very different set of pigments. In comparison to the FCP, the absorption spectrum of the F710 antenna has a diminished contribution of the red-shifted fucoxanthin absorbing above 500 nm as well as of the Chl *c* around 460 nm (Fig. 1C). The well-defined feature at ~490 nm results from increased contribution of diadinoxanthin. Overall, the F710 antenna exhibits significantly lowered content of both total carotenoids and Chl *c* relative to Chl *a*. This results in the region 450–600 nm contributing about 35% of the total absorption, in contrast to ~50% in FCP (computed as a ratio of the integral of the absorption spectrum over the 450–600 nm region to the integral of the whole spectrum, 400–750 nm). This decrease of absorption in the blue-green spectral window is compensated by a greatly enhanced Chl *a* absorption in the red/far-red region (~2 fold increase of the Chl *a* Q_y_ area). Interestingly, this represents a direct parallel to the red-light acclimation in *C*. *velia* ^24,31^, where the RL culture also expressed two spectrally distinct antenna types: (i) CLH, the usual light-harvesting complex that mainly contains an isofucoxanthin-like carotenoid absorbing strongly >500 nm, and (ii) the red antenna (red-CLH) harboring red Chl *a* forms in a higher oligomeric complex, enriched in the ‘bluer’ carotenoid violaxanthin. The differences in pigment composition between the standard and red-shifted types of antenna complexes (FCP vs. F710 and CLH vs. red-CLH) appear to minimize the spectral overlap between them. However, the difference in pigmentation might be also dictated by structural constraints preventing the fucoxanthin-binding LHC proteins from adopting the tertiary structure of the pigment binding pockets required to achieve pigment-protein and pigment-pigment interactions that are necessary to induce the ~30-nm red shift of the Chl *a* absorption.

**Figure 4:**
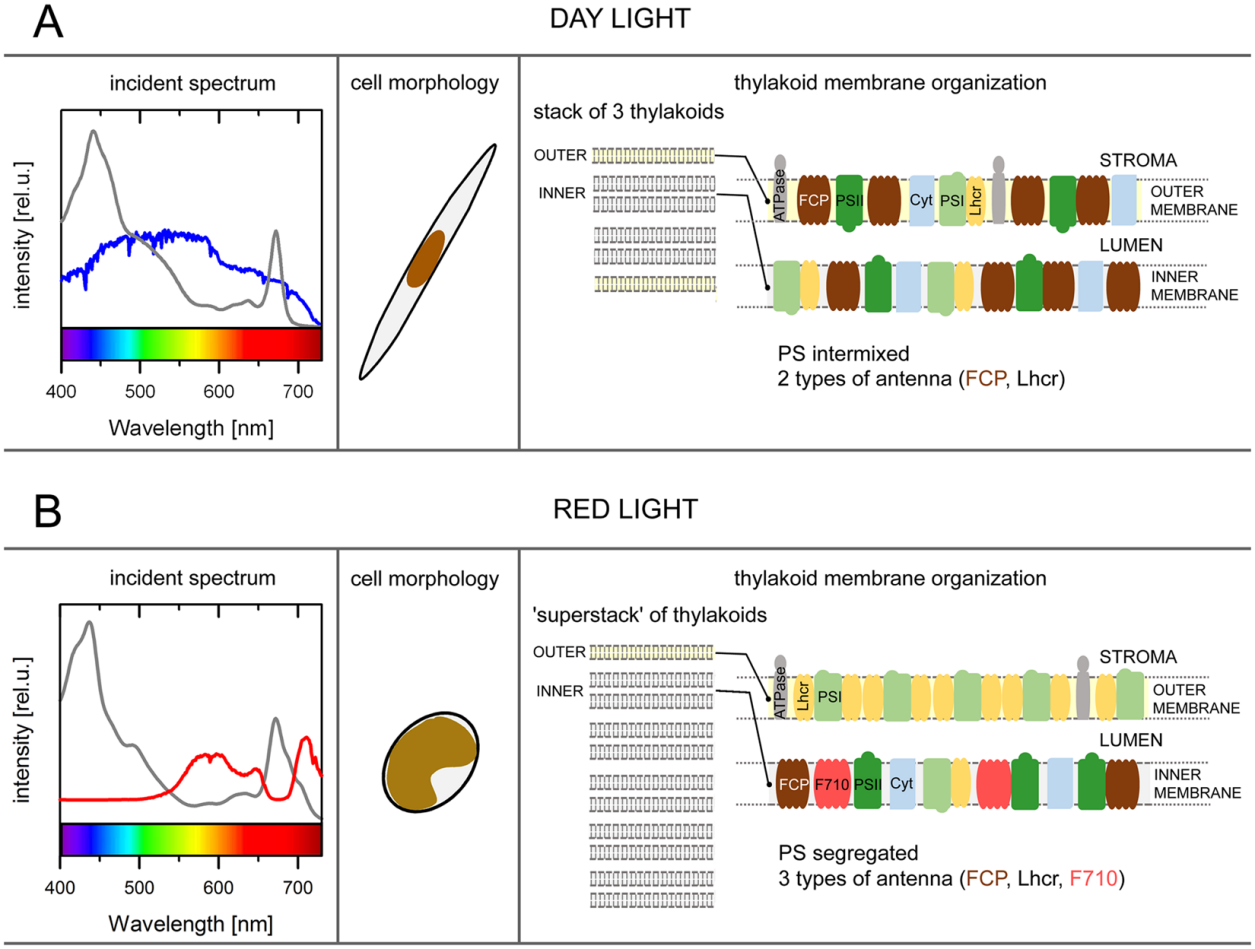
A model summarizing the differences in the thylakoid architecture and photosynthetic apparatus organization in the pennate diatom *P*. *tricornutum* acclimated to different quality of incident light, corresponding to (**A**) planktonic or (**B**) benthic conditions. Left panels show spectra of daylight (AM1.5 global-ASTM G173 in photons·m^-2^) filtered by water absorption (0.5 meter depth, *blue*) and daylight spectrum affected by water absorption as well as absorption of the photosynthetic pigments (*red*). Gray lines correspond to the absorption spectra of the typical light harvesting antennae (FCP or F710, respectively) adopted from Fig. 1C. Middle panels present dominant cell morphotypes while right panels show thylakoid membrane models based on current understanding of the chromatic acclimation in *P*. *tricornutum*. Under the DL conditions (**A**), cells are present mostly in the fusiform morphotype, the photosystems (PS) are randomly distributed on thylakoids organized in triplets, and light is harvested by two antenna types. In contrast, RL (**B**) induces i) formation of thylakoid “superstacks”; ii) photosystem I segregation. The PSI-rich regions (shown here) are devoid of other components of electron transport chain^27^; iii) production of an additional light-harvesting antenna with red-shifted absorption that consists of oligomers composed solely of the Lhcf15 protein. The acclimation to RL conditions is also accompanied by a marked change in the cell morphology.

The expansion of the absorption cross section in the wavelengths above 680 nm at the expense of the green spectral region suggests that the purpose of the red-shifted Chl *a* antenna complexes is the enhancement of light harvesting in the specific habitats with diminished availability of visible light such as bottom layers of stratified environments, shaded by upper layers inhabited by phototrophs, niches that have been known to be occupied by Cyanobacteria synthesizing unique red-shifted chlorophylls *d* and *f* ^36–38^. Hence, the red-shifted antenna was previously identified in the coral-endosymbiotic or epizoic algae *Ostreobium* and *C*. *velia* ^24,39–41^. Another example of a habitat where adaptation towards utilization of the far-red radiation might be beneficial are eutrophic waters, where the maximum of downwelling radiance can also lie above 700 nm^42^ due to combined optical effects of phytoplankton, water and suspended particulate matter. Given that red and far-red radiation is strongly absorbed by water, such adaptation would be expected to be limited to organisms inhabiting shallow waters. Illustration of the above points is given in the left panels of Fig. 4, where the quality of incident light in different aquatic environments is compared to absorption properties of the LHC of *P*. *tricornutum*. Figure 4A compares FCP absorbance with idealized underwater daylight spectrum modelled as standard AM1.5 global-ASTMG173 solar spectrum^43^ attenuated (intensity × transmittance) by 0.5 meter of water column (computed using published water absorption spectrum^44^); in Fig. 4B, the additional effect of shading was modelled by including the transmittance of DL culture *P*. *tricornutum* with density corresponding to OD = 0.05 (cm^−1^) at Chl *a* maximum, a realistic value for eutrophic waters^45^. This led to attenuation of the visible light and increase of prominence of the region around 700 nm, overlapping with the far-red tail of F710 Chl *a* absorption. A more quantitative comparison of absorptive properties of FCP and F710 is presented in Suppl. Fig. S5. The relative benefit of the F710 antenna versus FCP in an underwater environment with high phytoplankton content is shown in panel C of Suppl. Fig. S5.

While the computations used to produce spectra in Fig. 4 and Suppl. Fig. S5 are not meant to provide an exact model for the light penetration into the water column and serve as an illustration of the concept of the red-antenna function, they correspond very well to experimental spectra of the downwelling radiation in eutrophic coastal waters^42^. Since the habitats characterized by depletion of visible light are likely to be light-limiting in general, shared aspects of low light and red-light acclimation are to be expected. Hence, acclimation to red light in this case has to be viewed as a response to the lack of other photosynthetically usable wavelengths. The above given considerations imply, among other things, that algal cultures grown at regular daylight spectrum could develop, at least partially, features of the RL culture due to self-shading, particularly in an absence of stirring, allowing for stratification. Another functional aspect of the red-shifted Chl *a* that deserves attention is that of photoprotection. Presence of low-energy pigments acting as excitation traps can be expected to enhance efficiency of energy dissipation. Indeed, Lavaud and Lepetit^46^ showed a correlation between the fluorescence emission around 710 nm and the extent of the non-photochemical fluorescence quenching (NPQ), a photoprotective mechanism, in several diatom strains. Thus it is possible that presence of red-shifted LHC might be also advantageous for organisms inhabiting environments with fluctuating irradiance.

Compared to the spectroscopy and biochemistry of the Lhcf15-based F710 complex, the observations regarding modification of cell morphology do not yield easily to interpretation. In the work of De Martino *et al*. (2011)^30^, rounded cells were identified with the biofilm-forming culture while the fusiform cells were characterized as planktonic type. This offers an appealing parallel to the present observations of the RL culture, equipped by the red antenna for the presumed existence in the shaded environments. Moreover, although in our experimental setup the RL culture was continuously stirred, which prevented formation of a continuous biofilm layer, cells from the RL culture showed a tendency towards aggregation, consistent with the expected properties of biofilm builders. On the other hand, in the cited work, the rounded morphotype was also produced by cultures exposed to extended periods of abiotic stress and involved features of resting cells, including disorganization of the plastid. On the contrary, the rounded cells of our RL culture showed expansion of the membrane system^27^ (summarized in Fig. 4B) in a chloroplast occupying major part of the cell volume, a clearly different physiological response. Thus, rounded morphotype appears to be a very general response to adverse conditions and based on^30^ it can be expected that the oval and rounded morphotype represent endpoints of a continuum.

Hence the observed response of the RL culture encompasses three different levels of cellular organization: the rounded morphotype indicative of suboptimal growth conditions, expansion of thylakoid membrane system allowing for increased absorption cross-section, and the expression of the specific antenna to modulate the spectral coverage of the antenna system. Relative importance of different environmental factors for each of these processes is still to be investigated in detail. It is important to note that the oval morphotype, abundant in the ageing, and consequently likely nutrient-depleted DL culture, as well as slowly growing, low-light DL culture, did not show higher tendency to develop the red-shifted antenna under the experimental conditions used. Thus, in terms of the organization of the light-harvesting apparatus, the oval cells of the DL culture did not present a direct intermediate between the spindle-shaped cells and rounded cell types under red-enhanced illumination. This strengthens the interpretation of the RL-associated phenotype as a syndrome of specific acclimation mechanisms that have evolved to overcome particular ecological limitation as opposed to a generalized stress response.

## Conclusion

Integrating the recent findings, we propose a summary model for the low-light/red-light acclimation of the photosynthetic machinery in the pennate diatom *P*. *tricornutum* upon transition between the two light conditions (Fig. 4). In the planktonic DL phenotype, the chloroplasts retain the usual diatom triplet thylakoid organization and mostly random distribution of the photosynthetic complexes that utilize two oligomeric antennae for light-harvesting, i.e. the PSI-specific Lhcr antenna and the major Lhcf protein-based FCP antenna (Fig. 4A). In benthic or biofilm conditions, the light spectrum is filtered by cells occupying the water column but retains the far-red part around 700 nm. In the resulting phenotype, the chloroplasts remodel their thylakoids into superstacks while segregating the photosystems^27^, which is made possible by a flexible architecture of the diatom plastid^47^. To optimize light harvesting by trapping photons of longer wavelengths under RL conditions, a third oligomeric antenna F710 is synthesized in addition to both the Lhcr antenna and the major FCP antenna (Fig. 4B).

## Methods

### Culture conditions

As previously detailed in^25^, *P*. *tricornutum* cell culture (SAG collection, strain 1090–1a) was grown in a modified artificial sea water medium with Si and illuminated with a standard halogen lamp. Herein denoted as red-light (RL) conditions. The culture was maintained at 22 °C, under gentle constant aeration and stirring at light intensity of ~20 µmol photons m^−2^ s^−1^ in a 16/8h light/dark cycle. The cells were harvested on 8^th^ to 10^th^ day since inoculation when the F710 signal was fully developed in the RL culture even when culture was started from DL conditions. When the RL culture was maintained continuously by regular inoculation and nutrient addition the results were identical to those presented here. More frequent nutrient addition also did not alter the results but the cell suspension was then too diluted for protein purification purposes. Day-light (DL) culture was cultivated in the same manner as the RL culture except that a metal halide lamp as a source of day-light spectrum was used. The faster-growing DL cells were collected 3 to 4 days after inoculation. At both growth conditions, the fitness of the cells during cultivation was monitored by measuring Fv/Fm parameter, which for all studied cultures stayed in the range of 0.66–0.69. Growth rates were analyzed as in^25^.

### Light-harvesting antenna preparation

Membrane protein fractions enriched in red antenna were prepared as described previously^25^. In short, detergent-solubilized thylakoid membranes from the RL culture were sub-fractionated by ultracentrifugation on a linear sucrose density gradient (Supplementary Fig. S1A). Zone 5 of the sucrose gradient was subsequently subjected to gel filtration chromatography. The resulting chromatographic profile is shown in Supplementary Fig. S1B. In this study, we made use of the fraction 3, enriched in antenna proteins^25^ and preserving emission at 710 nm, for experimental work.

### Protein composition analyses

The antenna complexes from RL culture were separated with a colourless native polyacrylamide gel electrophoresis (CN-PAGE) on a 4.5–14% linear gradient polyacrylamide gel^48^. A total amount corresponding to 10 µg of chlorophyll was loaded per lane. The protein complexes were visible without gel staining and apparent molecular masses were estimated by co-electrophoresis of a high molecular weight protein standard (NativeMark^TM^, Novex, USA). Analysis of the pigment composition of the antenna complexes separated using CN-PAGE electrophoresis gels was performed using two different approaches: i) by HPLC analysis of pigment-protein complexes released from CN-PAGE gels and ii) by fitting of absorption spectra of the antenna bands excised from the gels. For HPLC analysis, the gel bands containing antenna complexes were excised, homogenised and immersed in 50 mM HEPES buffer, pH 7.5, supplied with 1% β-D-dodecylmaltoside. After 24 hour incubation, the extract was concentrated and analyzed by HPLC as described in^25^. Independent pigment content estimation was provided by fitting of the 420 nm–600 nm region of the absorption spectra of the antenna bands excised from the gels by spectra of the pigments in solution^49^. For the fitting, the absorbance spectra were first converted to wavenumber x-axis. Absorption spectra of pure pigments in the HPLC mobile phase were used as the fitting basis and respective extinction coefficients^25^ were used for quantification. The fits of FCP absorption from Premvardhan *et al*.^49^ were used as an initial guess for FCP, with 3 spectral pools of fucoxanthin, and 1 pool of Chl *a* and Chl *c* and diadinoxanthin, respectively. Analysis of the F710 spectrum started from the pigment set of FCP, by replacing the high-energy fucoxanthin pool with diadinoxanthin. Protein identification using tandem mass spectrometry (MS/MS) was carried out after denaturing SDS-PAGE in a second dimension, where the protein constituents of the complexes were separated based on their mass. A low molecular weight protein standard (Precision Plus Protein^TM^, Biorad, USA) was used to estimate the protein masses on the gel. MS/MS analysis was performed on a NanoAcquity UPLC (Waters, USA) online coupled to an ESI Q-TOF Premier Mass spectrometer (Waters, USA) as described in^50^.

### Spectroscopy

The bands containing antenna complexes were cut out from the CN-PAGE gel, placed in plastic cuvettes and directly used for the spectroscopic measurements. Room temperature absorption spectra were recorded using Shimadzu UV-2600 spectrometer (Kyoto, Japan). A mask for the semi-micro cuvettes was used to limit the passage of the measuring beam to a small spot within the homogenously pigmented part of the gel strips to eliminate distortion of the absorption spectrum. Fluorescence emission and excitation spectra were recorded at room temperature on a Spex Fluorolog-2 spectrofluorometer (Jobin Yvon, Edison, NJ, USA). The emission spectra were recorded in perpendicular geometry in the cuvette-mounted samples. The excitation and emission slits were set to 2 nm. The excitation spectrum of the F710 band was detected at 730 nm.

### Confocal laser scanning microscopy and image processing

Freshly collected RL and DL cells of approximately the same optical density (OD_750_ ~ 0.2) were used for confocal microscopy imaging. Images of *P*. *tricornutum* cells were obtained with confocal laser scanning microscope Zeiss LSM 880 (Jena, Germany) using a 63× oil-immersion objective and a resolution of 2048 × 2048 pixels. Chlorophyll autofluorescence was excited with a 488-nm argon laser, and fluorescence emission was detected at a bandpass of 650–696 nm (red region) and 705–735 nm (far-red region), respectively. To prevent cell movement, a droplet of the cells was covered with a round slice of agarose gel directly on a glass slide. Confocal images were processed using locally written MATLAB® (MathWorks Inc., USA) scripts. Images of individual chloroplasts were picked automatically from the confocal micrographs with a threshold at 4% of maximum intensity and the far-red/red channel ratio was computed for all individual pixels within the chloroplast image; the pixel-wise ratios were subsequently averaged over the whole area of the chloroplast. The dimensions of the algal cells were measured manually in the bright-field confocal images.

## Electronic supplementary material

Supplementary information
