# Supplementary material for: Red-light phenotype in a marine diatom involves a specialized oligomeric red-shifted antenna and altered cell morphology
Source: Sci Rep. 2017 Sep 20;7:11976. doi: 10.1038/s41598-017-12247-0 (PMC5607283; doi:10.1038/s41598-017-12247-0)
Supplement: Supplementary file 1 — Supplementary information [file 41598_2017_12247_MOESM1_ESM.pdf]

Supplementary data for

Red-light phenotype in a marine diatom involves a specialized oligomeric red-shifted antenna and altered cell morphology

Miroslava Herbstová, David Bína, Radek Kaňa, František Vácha & Radek Litvín[*]

[*] rlitvin@prf.jcu.cz

A **B**

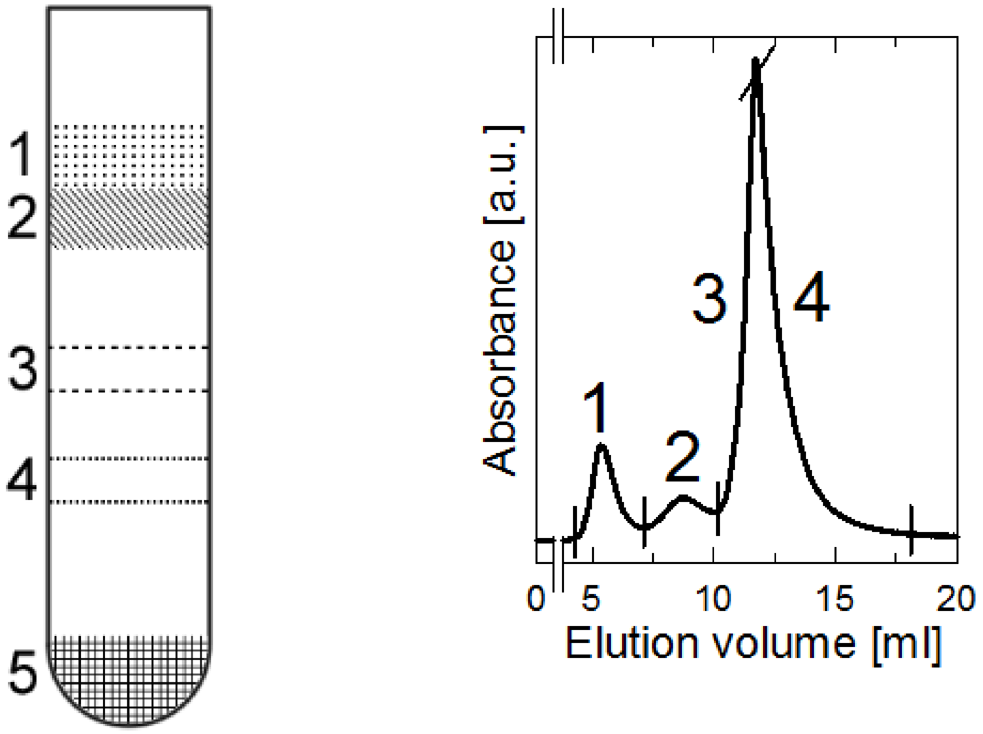

Supplementary Figure S1. A course of isolation and purification of the Lhcf-protein based oligomeric antenna complexes from *P. tricornutum* grown under red light (RL). **(A)** Schematic representation of the separation on a linear sucrose density gradient, and **(B)** gel filtration profile of the sucrose gradient zone 5. The antenna-rich fraction 3 preserving F710 emission at room temperature was used for further examination. The data shown here are adopted from a previous work (Herbstová et al. 2015, *Biochim. Biophys. Acta*, 1847:534-543) and are shown for reader's convenience.

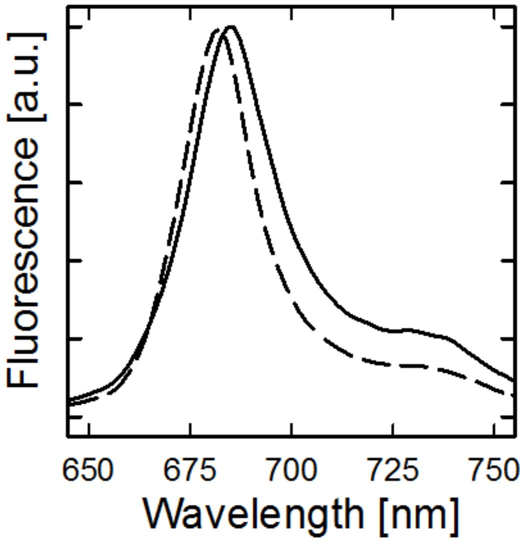

Supplementary Figure S2. Room temperature fluorescence emission spectra of the trimeric (*dashed line*), and the oligomeric (*solid line*) FCP antenna complexes from RL adapted *P. tricornutum*. The spectra of the antenna complexes separated by native electrophoresis were measured directly in the gel.

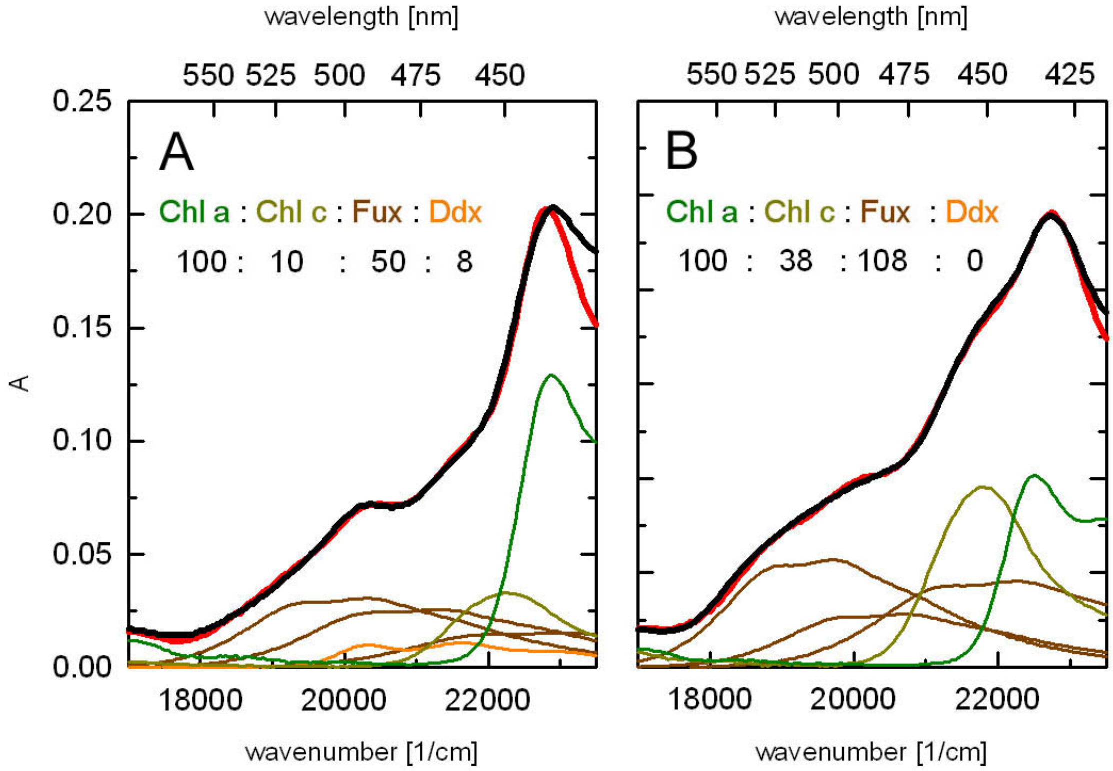

Supplementary Figure S3. Absorption spectra of F710 (**A**) and FCP (**B**) embedded in CN-PAGE gel, fitted by the spectra of pigments in solution. Measured spectra are plotted in black, fits are in red, pigments are color coded. Fux, fucoxanthin; Ddx, diadinoxanthin. Pigment stoichiometries derived from the fits are also shown. For comparison to results of HPLC analysis of the samples see main text.

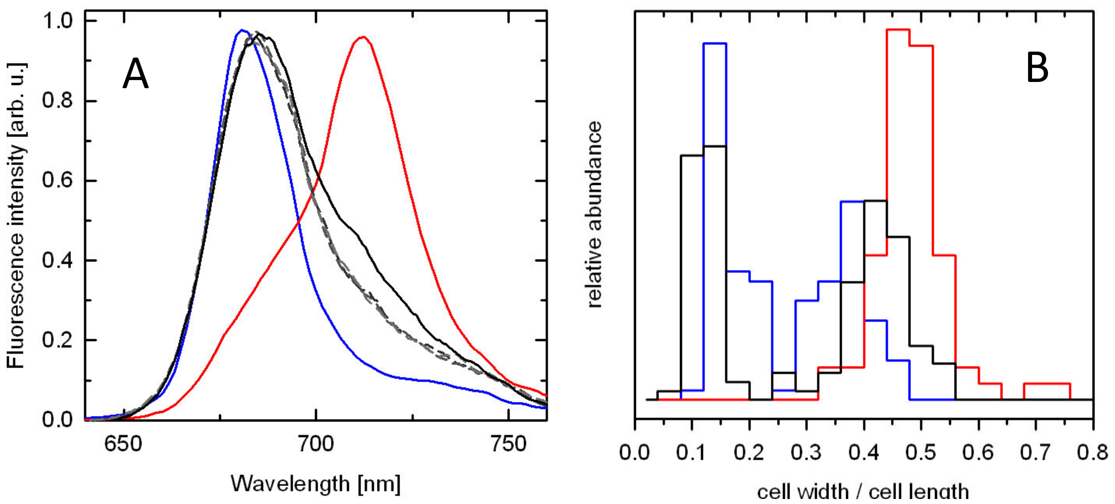

Supplementary Figure S4. Properties of the low-light DL culture with growth rate reduced to 0.16 day^{-1}. **A**: room temperature emission spectra of the ‘slow’ culture, black, compared to standard DL (0.5 day^{-1}), blue line and RL (0.16 day^{-1}), red line, cultures. Comparison to **Supplementary Fig S2** suggests that the difference in emission between the two DL cultures lies mainly in the increased abundance of oligomeric FCP in the ‘slow’ culture. Dashed lines denote spectra of the ‘slow’ DL culture on day 8, 11 and 15. Black line corresponds to slowly growing DL culture at day 25 after inoculation. Panel **B**: Comparison of cell dimensions between the three cultures. Colour coding is the same as in the left panel.

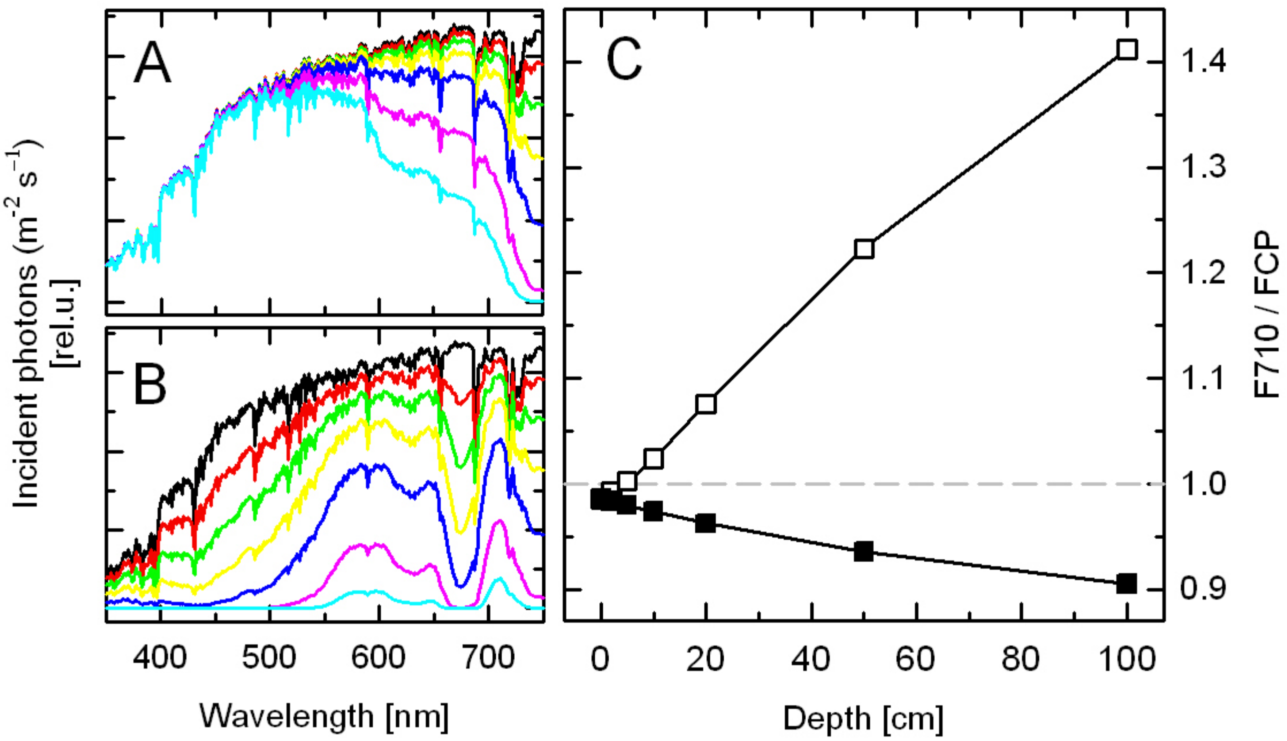

Supplementary Figure S5. **A, B**: Simulated underwater spectra at different depths ranging from 0-100 cm computed using AM1.5 global-ASTMG173 solar spectrum[43] attenuated (intensity × transmittance) by water column (computed using published water absorption spectrum[44]) and an additional effect of shading by phytoplankton. This was modelled by including the transmittance of *P. tricornutum* cells of DL culture corresponding to OD = 0.0005 (cm^{-1}, panel A) or 0.05 (panel B) to simulate different phytoplankton density; these are realistic values of regular (A) and highly eutrophic (B) coastal waters[42], respectively. For simplicity, the distribution of algal cells was assumed to be homogeneous through the water column. Specific assumed depths can be understood from symbol positions in panel C, cyan line is at 100 cm, pink is at 50 cm, dark blue at 20 cm, etc.

Panel **C** shows the comparison of light-harvesting efficiency of FCP and F710 antennas in environments characterized by the spectra in panels A and B. The points in panel C represent ratio (F710 / FCP) of spectral overlap of the two LHCs with incident irradiation spectra shown in panels A and B. Spectral overlap was computed by multiplying, at each wavelength, the incident irradiance (panel A or B) by the absorption of F710 or FCP (after normalizing the absorption spectrum to unity area). Then, this product calculated for each wavelength was integrated over the range 350-750 nm. Empty symbols correspond to the high-phytoplankton case (high shading, panel B), filled symbols to low phytoplankton (low shading, panel A). The F710 antenna becomes useful (F710 / FCP > 1) for light harvesting under highly shaded conditions. The same F710/FCP ratios calculated for growth lamp spectra used in this work are 1.25 (RL) and 0.99 (DL). Our growth illumination spectra thus correspond well to a shaded and non-shaded environment, respectively.

References are fully cited in the main text.

Supplementary Table 1. Peptides identified by MS/MS in 2D analysis (Fig. 2) of the spots corresponding to the F710 oligomer (spot # 1), and to the oligomeric (spot # 2) and trimeric (spot # 3, 4 and 5) FCP antenna complexes isolated from RL-grown *P. tricornutum*. Data are based on five biological replicates.

ID Name	Spot #	AA	Peptides	Start	End	Products	Score	MH+ Error
EEC50685.1 **Lhcf1**	2, 5	196	(K)DITGGEFVGDFR(N)	136	147	5	34.6	-0.0009
			(R)AIELNQGR(A)	168	175	8	367.5	-0.0038
			(R)NNYLDFGWDTFSEDK(K)	148	162	10	7.4	0.0011
EEC50684.1 **Lhcf2**	2, 5	198	(K)DITGGEFVGDFR(N)	136	147	14	1510.4	-0.0026
			(R)AIELNQGR(A)	168	175	10	588.3	-0.0045
			(R)LGGDIALDGTK(F)	90	100	13	1380.5	-0.0049
			(R)NGYIDFGWDSFDQETK(L)	148	163	11	616.5	0.0027
			(R)ISMLAVAGYLAQEAGWR(L)	73	89	3	0.5	0.0055
EEC50682.1 **Lhcf3/4**	2,4	198	(K)TFAEIPNGFAAFK(E)	100	112	11	673.2	-0.0002
			(R)LPGTIDYSGK(T)	90	99	11	362.2	-0.0027
			(R)AIELNQGR(A)	169	176	12	1904.3	-0.0057
			(R)NGAIDFGWDTFDEETQFK(K)	149	166	10	3.8	0.0103
			(R)DLTGEAEFVGDFR(N)	136	148	10	92.0	0.0006
			(R)ISMLAVVGYLVQEAGVR(L)	73	89	6	27.1	-0.0033
EEC44019.1 **Lhcf5**	2, 5		(R)NDFIDFGWDSFDEETK(M)	148	163	15	33.4	0.002
			(K)DITGGEFVGDFR(N)	136	147	14	1008.9	0,0001
			(R)AIELNQGR(A)	168	175	9	622.7	-0.0065
			(R)ISMLAVAGYLVQENGIR(L)	73	89	3	4.9051	0.002
EEC43939.1 **Lhcf6/7**	2, 4	204	(R)LPGTIDFAGTK(F)	92	102	14	654.7	-0.0011
			(R)QYNVELNQGR(A)	168	177	3	114.4	0.002
			(K)YIDFGWDSFSDEEK(A)	152	165	6	9.1	0.0045
			(R)DWVGGESVGDFR(N)	138	149	6	46.3	0.0045
			(R)FVELK(H)	148	163	1	0.7746	0.001
EEC45673.1 **Lhcf8**	2, 4	200	(K)LGVSILPDL(-)	192	200	14	1341.7	0.0096
			(R)LPGNIDYSGLK(F)	91	101	20	714.8	-0.002
			(R)AIELNQGR(A)	170	177	15	1393.5	-0.0076
			(K)FADVPGGFK(A)	102	110	9	728.6	-0.0064
			(R)DELGAQPPLGFFDPLGLVK(D)	36	54	25	1883.4	-0.01
			(A)DVPGGFK(A)	104	110	9	728.6	-0.0064
			(R)NGFIDFGWDSFDDATK(M)	150	165	9	317.1	0.0104
			(K)LGVSILPDL(-)	192	200	23	136.4	-0.0084
EEC44891.1	2, 3	200	(R)LPGNIDLSGTK(F)	90	100	17	943.6	-0.0021

**Lhcf9**								
			(R)DFVGGEFPGDLR(N)	136	147	5	21.4	-0.0018
			(K)FSDIPNGYAAIEAIPYAGK(L)	101	119	5	94.6	0.009
			(R)LVELK(H)	65	69	1	3.8	0.0042
			(R)NNYIDFGWDSFDDATK(A)	148	163	19	66.5	0.0037
			(R)TIELNQGR(A)	168	175	4	3.0	-0.0057
			(R)AAVVTGNFEEDIGATLPLGFWDPLGLVADGNQEK(F)	26	59	4	0.2551	0.002
EEC46120.1 **Lhcf10**	2, 4	199	(K)FTDIPGGFDALSAISK(E)	101	116	20	1390.4	-0.0009
			(R)LPGNIDYSGTK(F)	90	100	11	27.5	-0.0016
			(R)AIELNQGR(A)	168	175	17	2325.5	-0.0079
			(R)NDAIDFGWDTFDEETK(L)	148	163	4	1.0558	0.0131
			(R)GEFVGDFR(N)	140	147	3	0.8683	0.0048
			(R)YVELK(H)	65	69	4	115.5	-0.0041
EEC44018.1 **Lhcf11**	2, 4	197	(K)LGVSLIPN(-)	190	197	11	52.6	-0.0036
			(R)AIELNQGR(A)	168	175	7	16.6	-0.0019
			(K)DITGGEFPGDFR(N)	136	147	4	3.9	-0.0045
			(R)NDYIDFGWDSFDEETQFK(K)	148	165	7	0.9	0.0125
			(R)YVELK(H)	65	69	4	100.6	-0.0047
EEC45081.1 **Lhcf15**	1, 3	203	(R)LITGLPFLYN(-)	194	203	28	207.4	-0.0104
			(R)VAMLAVIGYIVPEFYR(F)	76	91	13	1893.1	0.0028
			(R)LAMLAFFELLR(H)	167	177	11	317.5	0.0037
			(R)FPGELMPGLAFK(D)	92	103	27	795.6	-0.0021
